# Chicago Medical Society

**Published:** 1869-04

**Authors:** 


### CHICAGO MEDICAL SOCIETY.

FRIDAY EVENING, January 29, 1869.

The Society was called to order, President Marguerat in the chair.

Secretary Macdonald read the minutes of the last meeting, which were duly approved, after slight modification by Dr. Davis.

The name of Dr. Bosley was proposed for membership by Drs. Seely and Clarke; also, Dr. W. M. Jackson, by Dr. Powell. Referred to Board of Censors.

Dr. Seely opened the discussion on the "Pathology and Treatment of Saccharine Diabetes." Is of the opinion that the term Glucohemia would be the best to signify the presence of sugar in the blood. Stated that it has been shown that the sugar formed in the liver is consumed by the lungs. Thinks sugar is also formed in the stomach; and the collection of sugar in the urine is due to its not being consumed by the lungs. Is of the opinion that the alkaline treatment is the best that has been advocated to diminish the amount of sugar in the urine. Has read some statements published by Mr. Day, in which he recommends the use of the peroxide of hydrogen. Cited one case which recovered in seventeen days.

Dr. Schmidt says, according to late experiments, it has been shown that there is no sugar to be found in the liver, nor hepatic veins, during life; or, at least, it cannot be detected upon immediate examination after life has become extinct. Other experiments showed, that if the femoral arteries were compressed, that sugar was formed; and hence they came to the conclusion that diabetes was due to paralysis of the nerves supplying the arteries, and a ferment in the blood. Has heard of cases treated successfully by the application of streams of water down the spinal column. Is of the opinion that arsenic is a valuable remedy in this disease.

Dr. Loverin asks if diabetes may not occur from nervous debility? If so, the treatment most appropriate would be tonics.

Dr. Davis remarked, that he had been very much interested by what had been said, and that he had never been satisfied with Bernard's experiments; and thinks that the more recent experimenters have failed to show or prove the source of the sugar, although they have thrown some light on the subject. He has noticed that punctures of the brain, or particularly of the medulla oblongata, have been followed by saccharine diabetes. Also, that strychnia and chloroform, used internally, have been followed by the same results. Says there was a doctor called on him that evening, who has been suffering from saccharine diabetes for some months, and who attributes the cause to the excessive use of strychnia.

Dr. Davis says, he does not think that nervous debility, nor even paralysis, can produce this disease, as we have a great many examples of great exhaustion from masturbation; also, paralysis and hemiplegia; and but very seldom any increase of urine. Hence, thinks it will not do for us to attribute the cause to debility of the nervous system. Thinks that the origin of the production of sugar is due to deficient action in some of the systemic and pulmonary capillaries. Says his confidence has been shaken in all remedies except such as promote assimilation. Hence, recommends the acetated tincture of calves' rennet, two drachms of which may be taken at each meal. Cited the case of a little girl who had saccharine diabetes some five years since, and who was successfully treated by "Haughton's Pepsin;" the patient not having shown any signs of the disease since. Has been in the habit of using liquid rennet, ʒj., dose before each meal, and

℞. Pulv. Opii,------------------------- gr. ss.,
Cupri Sulphus,--------------------- gr. $\frac{1}{6}$, after each meal, with good diet, avoiding starchy vegetables. Cited case of woman on West Side who was treated this way; and in two months hardly a trace of sugar could be found in urine, and has been entirely well for ssveral months. Noticed an article in the *New Orleans Medical Journal*, where bran bread and meat diet were highly spoken of, avoiding all starchy vegetables. Thinks we have yet to trace out the source of the sugar. Says if strychnine is capable of producing diabetes, it may still be beneficial in the treatment, if administered in medicinal doses. Cited cases where atrophy of the limbs was followed by an overdose of strychnine.

Dr. Paoli highly favors the vapor-baths in treatment of diabetes. Cited case of a woman who seemed to be benefited by the baths, but who, after a protracted illness, died.

Dr. Seely says that the pepsin has been used in France, but it is not considered a very efficient remedy. Thinks, with Dr. Davis, that nervous debility is not productive of the disease. Is of the opinion that the peroxide of hydrogen is worthy of trial.

Society next proceeded to Reports of Cases.

Dr. Paoli reported case of Swedish woman whom he was called to see at 10 P. M. last Saturday. The woman had been in care of a midwife, and was delivered of a small child at 3 A. M. Hemorrhage continuing from time of delivery until he was called. Found patient lying on her side; head high; pulse feeble, and greatly prostrated. Placed a pillow under her hips, and gave a teaspoonful of turpentine, and produced friction over abdomen. Pulse soon began to improve, and all hemorrhage ceased.

Passed to Miscellaneous Business.

Dr. Davis proposed next subject for discussion:—"What is the active agent that produces convulsions in uræmic poisoning, and the most reliable treatment?"

Drs. Schmidt and Paoli were appointed to open the discussion, two weeks hence.

Dr. Davis thinks it in the power of those present to make these meetings interesting, and suggests that every member read upon the subjects proposed for discussion, and be prepared to give their experience; thus benefiting each other.

Members present: — Drs. Marguerat, Macdonald, Davis, Paoli, Loverin, Grosbeck, Guerin, Fredigke, Schmidt, Seely, Wickersham.

Society adjourned.

---

FRIDAY EVENING, February 5, 1869.

The Society was called to order by the President, Dr. Marguerat.

Secretary Macdonald read the minutes of last meeting, which were duly approved.

The President remarked that there were two members to be elected to-night.

Dr. Paoli recommended that the election be postponed until next meeting.

The name of Dr. William T. Johnson was proposed for membership by Dr. Fisher.

Under call for Pathological Specimens,

Dr. Holmes presented an ossified crystalline lens, which had been shaking around loosely in the anterior chamber of the patient's eye for a period of five years. It was removed at the patient's request, as he had suffered considerable pain. The sight of the eye was lost, as it was the result of a blow.

Dr. Bogue asked if the anterior chamber was filled after the extraction?

Dr. H. says it was, preserving the rotundity of the eye.

Drs. Paoli, Quales, and Marguerat participated in the discussion.

Dr. Bogue presented a specimen, the result of conception. During the hemorrhage, the mass was partly held by the contraction of the os uteri. The sac was ruptured on his hand, and he says that he does not think there was anything but fluid escaped. Between the amnion and chorion found quite a large blood-clot. The question is, What has become of the fœtus? There was no appearance on either side of the attachment of the cord. Judges the patient to have been in about the sixth week of pregnancy.

Dr. Paoli asked if the fœtus did not pass off with the coagula?

Dr. Bogue was of the opinion that it was absorbed.

Dr. Fisher says he has seen a similar case, although rather larger, where there was no fœtus.

Dr. Marguerat also reported having removed a sac size of a pigeon's egg, containing a clear fluid, at the third month of pregnancy.

Dr. Bogue presented, for Dr. Hutchinson, a portion of a tape-worm, containing the head. Does not know what the Doctor used to cause its expulsion.

Dr. Paoli remarked, it was rare to see the head, but the tail is often seen.

Dr. Holmes asked if there were more tape-worms in this country now than common?

Dr. Trimble says that he has not seen many cases of late years.

Dr. Wanzer spoke of their being more common in warm climates—California. Said he lately expelled one from a man on the West Side, which the patient thinks was 200 feet long. The successful remedy was the ethl. oil of male fern, in 30-drop doses. Says he employed other remedies without benefit.

Dr. Bogue reported the case of a woman who had taken $\frac{1}{4}$ of a grain of atropia in solution, by mistake, supposing it was morphine. As soon as swallowed, there was a severe burning sensation in the stomach. Saw her in twenty minutes after, and gave an emetic, which operated immediately; in the mean time, there was considerable twitching of arms and legs, which began to pass away, together with the pain, in the course of half an hour. Next morning, the patient had some difficulty in seeing. The question is, Was it a poisonous dose?

Dr. Paoli thinks that a quarter of a grain would usually prove fatal, as the dose is from $\frac{1}{30}$ to $\frac{1}{60}$ of a grain.

Dr. Holmes also concurs in the opinion of Dr. Paoli. Related case of a lady who took $\frac{1}{2}$ grain, but vomited immediately after. He usually gives about $\frac{1}{100}$ grain at dose, internally. Thinks morphine and strong coffee the best antidotes.

Dr. Macdonald says that he gave a little boy of two years, who was suffering from incontinence of urine, $\frac{1}{12}$, increased to $\frac{1}{4}$ gr. ext. belladonna, for two weeks and a half, without symptoms of its toxic effect.

Dr. Bogue thinks, when a small portion of atropia is combined with morphia, adds very much to its anodyne influence, the effects continuing much longer than when the morphia is given alone. As an antidote, he prefers strong coffee to either whiskey or opium.

Dr. Bogue asked if $\frac{1}{4}$ g. ext. belladonna was not a pretty large dose for a child?

Dr. Marguerat says that Brown-Séquard records cases where he has given it in grain-doses in pertussis of children, having the effect to temporarily paralyze the fauces.

Dr. Paoli thinks it was commenced in $\frac{1}{8}$-grain doses, gradually increased to *one* gr.

Dr. Adolphus thinks it very dangerous to give one gr. at a dose.

Drs. Tucker and Wanzer participated.

Dr. Paoli spoke of having used the chloride of quinia in $\frac{1}{8}$-gr. doses in the treatment of five cases of malignant scarlet fever, with very beneficial results, reducing the pulse very rapidly. Asked the experience of the Society in its use.

Dr. Quales said he had heard it recommended in membranous croup.

Dr. Quales reported case of miscarriage. Patient was in hands of a midwife, and when he was called the hemorrhage was severe. The placenta was very large, and indurated. Fœtus some two months old.

Dr. Trimble reported a *rare* case of fracture of 5th metatarsal bone, from a sudden fall of the patient while skating. The sensation imparted to the young man at the time of fracture was that of an acute pain.

Dr. Bogue remarked, that fracture of the 5th metatarsal bone is of exceedingly rare occurrence, especially by turning of the foot.

Dr. Wanzer says he has rather a lengthy report on amputation of the shoulder-joint, but prefers to read it at the next meeting.

Society then proceeded to Miscellaneous Business.

Dr. Paoli presented copies of the Ohio and Illinois Laws governing the practising of physicians, which the Secretary read. Dr. P. then moved that a Committee of three be appointed to report on these laws at next meeting. The President appointed Dr. Davis Chairman, and Drs. Paoli and Trimble Associates.

Members present:—Drs. Knox, Marguerat, Macdonald, Davis, Bogue, Trimble, Paoli, Quales, Wanzer, Fisher, Tucker, Adolphus, Holmes.

Society adjourned.

---

Friday Evening, February 12, 1869.

The Society was called to order, President Marguerat in the chair.

Secretary Macdonald read the minutes of the last meeting, which were duly approved.

The Board of Censors reported favorably in the cases of Drs. Bosley and Johnson. Society then proceeded to ballot, Drs. Bosley and Johnson being duly elected as members of the Society.

Under call for pathological specimens, Dr. Fenn presented a salivary calculus extracted from Wharton's duct.

Society next proceeded to the discussion of the .subject chosen at last meeting, *viz.:* "What is the active agent that produces convulsions in uremic poisoning, and the best treatment?"

Dr. Schmidt opened the discussion, by saying that he had gone through a great deal of literature on the subject, and has found that most European authors refer to American authority, especially Dr. W. A. Hammond's work; but says that experiments have shown that not a single substance of the urine would produce uremia except urea, which had to be injected in very large quantities in order to produce the symptoms.

Says he has a patient under treatment at present at the Jewish Hospital, who has some fifteen fistulas, the urine flowing from all. He opened the fistula and introduced a cathcter. Patient had chills and headache, which symptoms were mitigated for several days by the administration of hydrochloric acid.

Dr. Paoli says that uremia exists in albuminuria, and is ocsioned by the blocking of the ureters. Askcd Dr. Schmidt if he tested the urine for albumen? Dr. S. replied that he had not.

Thinks albumen is present in the urine of nearly every pregnant woman at the 7th or 8th month.

Dr. Paoli says he now has a case presenting symptoms of uremic poisoning. The patient is an old lady, and her bladder does not contain more than a tablespoonful of urine. Specific gravity, however, is about normal. Has been giving nitromuriatic acid, which seems to give some relief.

Cited a case in cholera-time of a young man who did not pass any urine for four days, still there were no symptoms of uremia. Dr. Trimble saw the same case. Patient died in a comatose state. Thinks it is evident that urea is found in the kidney, and not in the blood.

Dr. Schmidt says he was called some ten years since in consultation with two other doctors. Patient was a man of 35, and had been suffering from amaurosis. When he arrived, found the patient in a dying condition. Had been gradually losing his sight for three weeks, but had continued to work up to within two days. Pulse was weak, but all the functions seemed to be normal. Found a large tumor on the right side. Asked his wife in regard to the voiding of his urine. She said when he became excited, he would pass urine a half an hour at a time. Patient died that night. Found the right kidney to consist of a sac as large as my hat, while the left was very much enlarged, and fatty.

Dr. Gray reported the case of a young man who injured his hand. Three weeks after, tetanus set in. Applied turpentine and belladonna to spine by means of a cloth being saturated, over which he placed a warm flat-iron. Gave internally chloroform gtt. x. every fifteen minutes, during the time of which there was no spasm. Then resorted to bromide of potassium, in 20-grain doses, every two hours, without effect, as the spasms again recurred. Patient died in three days; and, although he had not passed water or anything from his bowels for 24 hours, upon the introduction of the catheter his bladder was found perfectly empty.

Dr. Fredigke says he has noticed an account of five cases of tetanus in the *London Lancet*, four of which recovered under the use of Calabar bean.

Dr. Foster remarked, that there is a horse-doctor in the city who gives hydrocyanic acid, in from one to two drachm doses, in the treatment of tetanus in horses. Also, tincture of aconite-root, in 20-drop doses, with very good success.

The Secretary then read the report on the Medical Bill, and it was moved and seconded that the bill be accepted.

Dr. Wickersham said that the bill presented was free from the objections of the one he opposed. Thinks it will start legislation for the protection of the people, and moves it be adopted, although it is borrowed from the Ohio bill.

Dr. Paoli says that he is sorry that the Society did not appoint Dr. Wickersham Chairman of the Committee to report on the bill, and says that the reason for adopting the Ohio bill is because it is more practical than the one originally proposed.

Dr. Reid thinks it necessary to appoint a Board to examine those who desire to practise, and recommends the appointment of separate Boards, Homœopathic, etc., to examine those of their own school.

Dr. Trimble says he thinks the remarks of Dr. Reid very appropriate, in order to make the bill more practicable.

Dr. Wickersham says he does not favor any such project.

Dr. Davis says that what ought to be done is the appointment from the profession of a Board whose duty it would be to examine every person desiring to practise medicine, diploma or no diploma; and says there was a period when two-thirds of all the States of the Union had such organized Boards. Thinks, however, if we ask for a Board, appointed by competent authority, the Legislature would not pass any such laws.

If the Board were appointed by the Governor, it would be nothing but a football in politics, and the Board would be made up of anything but what we want; and it would amount to about the same if the judges of courts had the appointment of Boards. Hence, does not believe there is any use in going outside of the profession for a Board. Not being able to do this, we next aim to have educated men in the profession. Thinks that if the Society do anything, they would do well to adopt the law, and recommends that a Committee of three be appointed to get the bill put in print, and send it to the Legislature, with the request that they accept this bill in place of the one proposed, as the opinion of this Society.

Dr. Davis recommended subject for discussion two weeks from to-night—

"Pathology and Treatment of Scarlet Fever."

Drs. Tucker and Guerin appointed as disputants.

Members present—Drs. Marguerat, Macdonald, Davis, Paoli, Bogue, Wickersham, Schmidt, Trimble, Reid, Tucker, Ray, Fisher, Loverin, Quales, Hutchinson, Baxter, Gray, Bridge, Foster, Guerin, and Fredigke.

Society adjourned.

